# Applying a Mixed-Method Approach to Improve On-the-Job Learning and Job Satisfaction in a Cohort of Interns at a University Hospital

**DOI:** 10.7759/cureus.15905

**Published:** 2021-06-24

**Authors:** Amna S Butt, Muhammad S Shamim, M Asghar Ali, Farah Qamar, Irum Q Khan, Swaleha Tariq, Syeda Amrah Hashmi, Quratulain Hafeez, Muhammed Tariq

**Affiliations:** 1 Gastroenterology, Aga Khan University Hospital, Karachi, PAK; 2 Neurosurgery, Aga Khan University Hospital, Karachi, PAK; 3 Anaesthesiology, Aga Khan University Hospital, Karachi, PAK; 4 Pediatrics, Aga Khan University Hospital, Karachi, PAK; 5 Emergency Medicine/General Surgery, Aga Khan University Hospital, Karachi, PAK; 6 Family Medicine, Aga Khan University Hospital, Karachi, PAK; 7 Medicine, Aga Khan University Hospital, Karachi, PAK; 8 Internal Medicine, Aga Khan University Hospital, Karachi, PAK

**Keywords:** medical education, quality improvement, medicine, internship, audit

## Abstract

Introduction: Job satisfaction is vital for the optimal functioning of medical practitioners. Herein, we report our experience of restructuring the internship program by identifying the gaps, developing, implementing strategies to overcome gaps and sharing the results of the pre-implementation and post-implementation audit, as an example for establishing a system for improving intern’s work-based learning and satisfaction in a university hospital setting.

Methods: Using Kern’s six-step instructional model, a prospective mixed-method study was conducted at Aga Khan University Hospital. In phase 1 (2013) gaps were identified by evaluating various aspects of the internship program. Strategies were developed and implemented to overcome the identified gaps. In phase 2 (2014-2016) the impact of these developmental strategies was assessed.

Results: A total of 65 interns, 30 residents, and 22 faculty members participated in phase I, while 71 interns participated in phase II. The reformation of orientation sessions, including practical exposure and content of sessions, opportunities to enhance hands-on experience and supervision in inpatient areas, operating rooms, supervision by fellows, supervision for hands-on procedures, career counseling, and mentorship, led to significant improvement in satisfaction. It was identified that the lack of hands-on opportunities can be overcome by surgical skills-based workshops. These reforms led to an overall rise in intern satisfaction (50% vs 75.4%, p=0.02).

Conclusion: Periodic restructuring of an existing program helps to improve the work-based learning experience and overall satisfaction among interns. This not only maximizes learning but also eases interns into their postgraduate life and workload subsequently enabling them to become more competent and well-rounded health practitioners.

## Introduction

Job satisfaction among hospital employees has been a topic of extensive research [1]. Lower job satisfaction rates have been associated with stress, burnout, perceptions about the workplace, and have been shown to affect not just physicians’ health and quality of life, but also patient satisfaction, patient care, and patient safety [1-8]. Mentoring has been shown to improve job satisfaction, especially for trainee physicians [9-11]. The Aga Khan University Hospital (AKUH) is one of the largest tertiary care hospitals in Pakistan offering a one-year structured internship program where interns are rotated in various disciplines. The Internship (House Job) Committee at AKUH recently revamped its program, based on an elaborate external review. The committee adopted the six-step approach described by Kern as an instructional design process to carry out this assignment [12]. The process started with an audit of the existing program, the actual restructuring that involved introduction and adherence to work hours, improving the orientation program, improving the content of teaching sessions, introducing hands-on simulator-based training, improving clinic and operating room exposure, introducing open forums for faculty-intern discussions, introducing opportunities for career counseling and mentoring, etc. This was followed by another audit to assess the impact of the restructuring. Herein we report our experience with this process and share the results of the pre-implementation and post-implementation audit, as an example for establishing a system for improving interns' on-the-job learning and job satisfaction in a university hospital setting. We aim to identify the gaps in the internship program at the Aga Khan University Hospital, to develop and implement the strategies to overcome the identified gaps, and to evaluate the impact of these strategies in improving the quality of the internship program over the next three years of implementation.

## Materials and methods

Study design and setting

This was a prospective mixed-method study conducted at AKUH in two phases during 2013-2016. The study was designed to identify the gaps in the internship program at AKUH and to develop and implement the strategies to overcome the identified gaps (phase I), and to evaluate the impact of these strategies in improving the quality of the internship program after one year of implementation (phase II).

The study was approved by the hospital Ethics Review Committee of AKUH, approval 4327-MED-ERC-16. The study participants were the interns, residents working in various subspecialties, program directors, and coordinators of various disciplines. The study was conducted in two phases. In phase I, to identify the gaps, interns who had graduated from the internship program in the preceding two years (2011-2012), as well as the interns who were enrolled during the year 2013, residents working in various subspecialties, program directors, and coordinators of various disciplines were surveyed using a cross-sectional study design through a standardized questionnaire. At the end of phase I, based upon feedback from study participants, strategies were designed and implemented. During phase II the impact of implemented strategies on the quality of the internship program was measured by using a “single-arm cohort” study design. This was done by approaching the interns subsequently enrolled in the internship program for the next three years (2014-2016) using the same questionnaire filled out by the interns in phase I. All these questionnaires were standardized for objective measurement of responses and were pre-tested before administration. The internal consistency of each questionnaire was evaluated by calculating Cronbach’s alpha.

Data collection and enrollment

**Figure 1:**
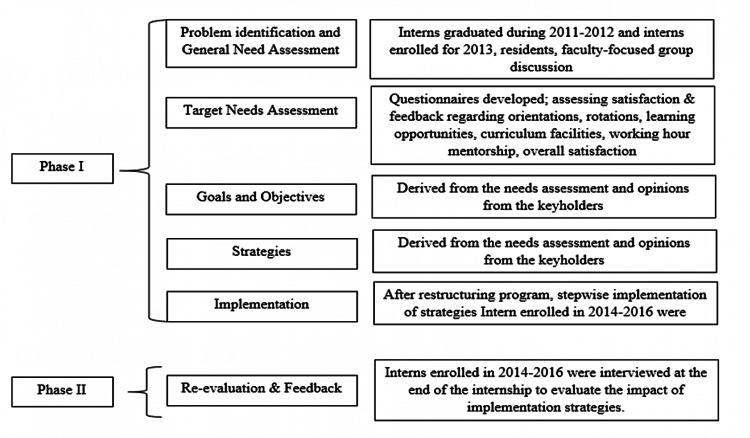
Study methodology

To evaluate and identify the gaps in various aspects of our internship program and to evaluate the impact of strategies, the Kern six-step model [13] was used, and the steps shown in Figure 1 were followed.

Details of internal and external reviews conducted in 2012 were reviewed. During phase I (2013) focus group discussions were carried out with small groups of interns, residents, and faculty members. Separate questionnaires were designed for interns, residents and faculty members, focusing on three broad categories: 1) orientation of the internship program 2) intern’s sub-specialty rotations 3) on job facilities and overall quality of the program. These questionnaires had both open-ended and closed-ended questions and utilized a 7-point Likert scale for ratings. The questionnaires were pre-tested and their internal consistency calculated through Cronbach’s alpha was 0.832, 0.971 and 0.981 for interns’, residents’ and faculty’s questionnaires respectively.

Gaps were identified and potential areas for improvement were prioritized. Strategies were developed to overcome the gaps and after necessary approvals, these strategies were subsequently implemented. The program was re-evaluated at the end of the year for the next three years, i.e. 2014-2016, using the same questionnaire.

Data was entered and analyzed by using SPSS version 22 (IBM Corp., Armonk, NY, USA). Frequencies (%) were calculated for categorical variables and mean ± SD for quantitative variables. The responses were measured using a Likert scale on an ordinal rating scale value from 1 to 7 and were further converted into two nominal categories of ≤4 and >4, which were represented in terms of frequencies and proportions. A cut-off value ≤4 was considered as “not satisfied” and >4 as “satisfied”.

To measure the difference in the quality of the program after implementing the strategies, the ordinal response data of phase I and phase II interns were compared by the Mann-Whitney U test. A p-value of <0.05 was considered significant. Qualitative assessment was done for open-ended questions and all responses to open-ended questions were transcribed for qualitative analysis. After reviewing all the responses, a coding frame was constructed. Topics in these responses were identified and categorized into themes and analyzed as such by calculating responses in percentages.

## Results

A total of 65 interns, 30 residents, and 22 faculty members participated in phase I (pre-implementation) and 71 interns participated in phase II (post-implementation). Among interns, 64% were graduates of private-sector medical colleges. The mean age of interns was 24.8 ± 1.1 years and 68.4% were female. Most of the interns aimed to join the AKUH internship program due to the high impact of the institution with training facilities matching international standards, structured programs with a secure workplace, and good salary structure. The responses of participants in phase 1 are described in Table 1. The qualitative analysis of responses comparing phase 1 and 2 is described in Table 2.

**Table 1:** Responses of faculty, residents and interns on a 7 point Likert Scale (Phase I) PGME =Post Graduate Medical Education,

	Faculty (N=22) n(%)	Residents(N=30) n(%)	Interns (Phase 1) (N=65) n(%)
Was the orientation provided at the beginning of your internship helpful?			
≤4	6 (27.3)	11 (36.7)	34 (52.3)
>4	14 (63.6)	17 (56.7)	28 (43.1)
No response	2 (9.1)	2(6.7)	3 (4.6%)
Were you satisfied with the specialty combinations offered to you at the start of internship?			
≤4	3 (13.6)	7 (23.3)	29 (44.6)
>4	14 (63.6)	19 (63.3)	33 (50.8)
No response	5 (22.7)	4 (13.3)	03 (4.6)
Was your rotation in compliance with the objectives?			
≤4	8 (36.4)	8 (26.7)	21 (32.3)
>4	7(31.8)	8 (26.7)	14 (21.5)
No response	7 (31.8)	14 (46.7)	30 (46.2)
Were you satisfied with the exposure and learning opportunities offered to you in the outpatient clinics?			
≤4	11 (50.0)	8 (26.7)	34 (52.3)
>4	8 (36.4)	15 (50.0)	29 (44.6)
No response	3 (13.6)	7 (23.3)	02 (3.1)
Were you satisfied with the exposure and learning opportunities offered to you in the inpatient areas/wards?			
≤4	5 (22.7)	6 (20.0)	22 (33.8)
>4	14 (63.6)	18 (60.0)	42 (64.6)
No response	3 (13.6)	6 (20.0)	01 (1.5)
Were you satisfied with the exposure and learning opportunities offered to you in the operating room?			
≤4	10 (45.5)	8 (26.7)	34 (52.3)
>4	4 (18.2)	7 (23.3)	06 (9.2)
No response	8 (36.4)	15 (50.0)	25 (38.5)
Are you satisfied with learning opportunities provided to you by your Residents?			
≤4	9 (40.9)	7 (23.3)	24 (36.9)
>4	12 (54.5)	17 (56.7)	39 (60.0)
No response	1 (4.5)	6 (20.0)	02 (3.1)
Are you satisfied with learning opportunities provided to you by your fellows?			
≤4	8 (36.4)	13 (43.3)	23 (35.4)
>4	8 (36.4)	7 (23.3)	39 (60.0)
No response	6 (27.3)	10 (33.3)	03 (4.6)
Are you satisfied with learning opportunities provided to you by your consultants?			
≤4	9 (40.9)	15 (50.0)	26 (40.0)
>4	12 (54.5)	9 (30.0)	38 (58.5)
No response	1 (4.5)	6 (20.0)	01 (1.5)
Were you satisfied with opportunities and supervision for hands on procedures?			
≤4	15 (68.2)	13 (43.3)	43 (66.2)
>4	6 (27.3)	14 (46.7)	20 (30.8)
No response	1 (4.5)	3 (10.0)	02 (3.1)
Were you satisfied with the CONTENT of mandatory sessions arranged by PGME?			
≤4	7 (31.8)	13 (43.3)	19 (29.2)
>4	13 (59.1)	12 (40.0)	45 (69.2)
No response	2 (9.1)	5 (16.7)	01 (1.5)
Were you satisfied with the on job facilities provided to you?			
≤4	6 (27.3)	10 (33.3)	19 (29.2)
>4	16 (72.7)	16 (53.3)	45 (69.2)
No response	00	4 (13.3)	01 (1.5)
Are you overall satisfied with your internship?			
≤4	8(36.4)	11 (36.7)	15 (23.1)
>4	13 (59.1)	15 (50.0)	49 (75.4)
No response	1 (4.5)	4 (13.3)	01 (1.5)

**Table 2:** Qualitative analysis of various aspects of the internship program {n(%)}

Categories	Comments	Interns Phase I	Interns phase II		Faculty	Residents
Theme 1: Reasons for choosing the Internship program at AKUH						
Institutional Impact	A globally reputable institution with diverse and efficient teaching and training facilities, matching international standards, better Salary, JCIA accreditation, Biggest PGME program in Pakistan, supervise hands-on practice	43 (50.6)	25(36.2)		-	-
Environment	Competitive yet secure working environment, better facilities	08 (9.4)	06(8.7)		-	-
Structured internship program	Quality training, opportunities for clinical, research training, well equipped with technology and matches with the international standard working system, link to various international health organizations, quality patient care, well-formed opportunity to learn new diagnostic/treatment modalities, practice evidence-based medicine.	11 (12.9)	12(17.4)		-	-
Aspiration	Become a part of alumni, gain comprehensive clinical experience, work in different specialties, avail career opportunity in AKU in future	15 (17.6)	18 (26.1)		-	-
Convenience	Previous work experience at AKU, Close to Home, AKU Graduate, Peers recommendations	08 (9.4)	08(11.6)		-	-
Theme 2: What was lacking in the orientation program?						
Practical exposure and content	Didactic, lengthy lectures and presentations, limited opportunities to learn hands-on skills in clinical areas including operational software e.g. online pharmacy system, maintaining medical records, ordering labs, making discharge summaries. The duration of the orientation program was too short to learn so many details Lack of guidance regarding the use of common medicines and interaction with patients, communication, and handing taking overs, generic pieces of information e.g important extensions. Lack of clarity regarding roles of an intern, working hours and specialty wise objectives were not defined Lack of opportunities to select subspecialty rotations during orientation Date and timings of orientation were overlapping with exams in other institutes thus lowering attendance No BLS/ACLS & basic surgical skill workshop scheduled in the initial days	49(83.1)	42 (66.7)		27(77.1)	28(77.8)
On site orientation	Disorganized/incomplete round of different wards/clinic areas/laboratory/radiology/ operating room	4(6.8)	05 (7.9)		03(8.6)	04(11.1)
Theme 3: What was helpful in the orientation program?						
Sessions and facility tour	Handouts, presentations, introductory speeches by faculty, career counseling, sessions related to common diseases, pharmacy, medical record documentation, briefing regarding guidelines & protocols, policies & ethics in patient care, Campus tour was helpful A reasonably organized orientation program for interns, information shared was useful.	53(66.3)	50(71.4)		20(66.7)	18(58.1)
Hands-on training	Acquaintance with use of CPOE system, overlap time with rotating interns & exposure for documentation work and interaction with patients, familiarization with learning resource center/IT/Library.	27(33.8)	20(28.6)		10(33.4)	13(41.9)
Theme 4: Which specific combinations of rotations do you feel should be modified?						
Medicine & allied	Rotations in medicine & subspecialties should be for 6 months Rotation in family & (ER), ICU should be made compulsory, combine relevant rotations Add radiology, nephrology and increase slots for Pediatrics and psychiatry Oncology should be removed	27(48.2)	21(46.7)	03(30.0)		05(33.3)
General Surgery(GS) and allied	GS should be a 3 months rotation as most countries require work experience of at least 3 months in GS and Medicine GS and subspecialty rotations are hectic and combination of rotations offered needs modifications Change 3 months Obs & gyne-pediatrics rotation to 2 months. Anesthesia, ENT, Obs/Gyn. + Pediatrics-Surgery combination should be offered in more combinations	26(46.4)	22(48.9)	05(50.0)		05(33.3)
Exposure	Every rotation should be of one month to have a diverse exposure PGME should send a list of rotating Interns for the whole year to the chief resident before the start of the internship Each rotation should be decided a priori & interns should be allowed to choose their specialty by themselves The rotations in private wing should be kept in mid or end of the internship because patients are too demanding.	03(5.4)	02(4.4)	02(20.0)		05(33.3)
Theme 5: What would you suggest to improve the shortcomings?						
Structure of rotations and learning objectives (Los)	Intern’s centric schedules allowing rotations in all subspecialties The outpatient, daycare and OR exposure should be mandatory, cut down on clerical work Discuss LOs of each rotation in beginning, make rotations more organized, add more academic and feedback sessions Develop mechanisms to improve the hands-on experience and procedural skills in wards and theaters Establish the end of rotation assessments	38 (64.4)	40 (74.1)	12 (85.7)		08 (61.5)
Workload balance and supervision	Protected time to attend academic sessions adequate breaks for rest Increase the number of interns to decrease workload, increase compliance with working hours A division plan for work should be made at the start of the surgery rotation Develop mechanisms for direct supervision by faculties, mentorships and regular feedbacks	21 (35.6)	14 (25.9)	02 (14.3)		05 (38.5)

Orientation sessions

Before the commencement of the internship, the PGME arranges a three-day mandatory orientation session for interns, for which they are paid as well. The utility of these sessions was assessed (Tables 1, 2, 3). The overall response rates were >90% and 43.1% of interns satisfied with orientation sessions during phase I. Based on the feedback received during phase 1, several changes were made in the orientation session. These include increasing duration from two days to five days, expanded exposure in clinical areas with hands-on experiences, the introduction of an ‘overlap’ between the outgoing and incoming interns, allowing smoother handover and continuity of care, and creating opportunities to familiarize interns with operating systems and policies. When the satisfaction levels for these orientation sessions were compared among phase I and phase II, the overall mean rank scores were much higher in phase II (Table 3). A higher proportion of interns identified gaps in the practical exposure and content of orientation as compared to phase 2 (83.1% vs. 66.7%). Another common reason for the low satisfaction rate was the lack of usefulness of sessions and facility tours. The satisfaction rates improved from 56.3% in phase I to 71.4% after restructuring in phase II (Table 2).

**Table 3:** Comparison of interns responses in Phase I and Phase 2 (pre- versus post-implementation of strategies to reconstruct internship program) measured on a 7-point Likert scale

Categories	Pre- Implementation (phase 1)				Post-Implementation (phase 2)				p-value
	Mean Rank	Percentile (Liker score)			Mean Rank	Percentile(Liker score)			
		25^th^	50^th^	75^th^		25^th^	50^th^	75^th^	
Was the orientation provided at the beginning of your internship helpful?	64.75	4	4	5	68.96	4	4	5	0.51
Were you satisfied with the specialty combinations offered to you at the start of the internship?	63.59	4	5	6	62.42	4	5	5	0.85
Was your rotation in compliance with the objectives?	40.99	3	4	5	48.37	4	5	5	0.18
Were you satisfied with the exposure and learning opportunities offered to you in the outpatient clinics?	63.02	2	4	6	71.48	3	5	6	0.20
Were you satisfied with the exposure and learning opportunities offered to you in the inpatient areas?	59.98	4	5	6	74.38	4	6	7	0.02
Were you satisfied with the exposure and learning opportunities offered to you in the operating room?	42.21	1	1	3	63.09	2	3	4	0.01
Are you satisfied with the learning opportunities provided to you by your Residents?	65.85	4	5	6	68.96	4	5	6	0.63
Are you satisfied with the learning opportunities provided to you by your fellows?	72.88	4	5	6	59.82	3	4	5	0.04
Are you satisfied with the learning opportunities provided to you by your consultants?	72.64	3	5	6	62.80	3	4	5.25	0.13
Were you satisfied with opportunities and supervision for hands-on procedures?	60.64	3	4	5	72.72	3	4	5	0.04
Were your working hours in compliance with the AKU policy of 80 hours/week averaged over 3 months?	65.92	1	2	4	66.07	1	3	4	0.98
Were you satisfied with the CONTENT of mandatory sessions arranged by PGME?	57.02	4	5	6	67.40	5	5	6	0.09
Were you satisfied with the on job facilities provided to you?	65.99	4	5	6	69.81	5	5	6	0.55
Are you overall satisfied with your internship?	60.31	5	5	6	74.93	5	5	6	0.02

Rotations in various subspecialties

The PMDC requires each intern to rotate in subspecialties related to Medicine and Surgery for the duration of six months each. Institutions can adjust rotations in a given domain as per availability. The AKUH offered a fixed rotation schedule with several combinations of sub-specialties. However, such preset combinations were not offered in medicine. In phase 1 approximately 63% were satisfied with the available combinations of rotations and almost one-third of interns found rotations in compliance with objectives. Hence, restructured 12 months of rotational plans were implemented consists of various combinations of rotations that interns can choose during orientation based on their admission test merit. The learning objectives of all rotations were developed and shared beforehand. Although the difference was not statistically significant, the mean rank score was higher for compliance with objectives in phase II.

The satisfaction level was low for learning opportunities in phase I. Hence, in each rotation opportunities were created to enhance hands-on experience and supervision. A statistically significant improvement in satisfaction was noticed for inpatient areas (59.9 vs 74.3, p=0.02), operating rooms (42.2 vs 63.0, p=0.01), supervision by fellows (72.8 vs 59.8, p=0.04) and supervision for hands-on procedures (60.6 vs. 72.7, p=0.04) (Tables 2, 3).

Based upon feedback in phase I, the opportunities that were created for career guidance, mentorships and contents of academic sessions were revised. The proportion of interns satisfied with opportunities provided for career counseling and mentorship was higher in phase II as compared to phase I (93.2% vs 69.8%). Though the mean rank score for the revised content of mandatory sessions was higher in phase II (67.4 vs 57.0), the difference was not statistically significant. A similar observation was found in the case of facilities at the workplace and compliance with the AKU policy of 80 hours/week averaged over three months.

Surgical skills workshop

Considering gaps in teaching basic surgical skills, simulation-based surgical skills workshops were conducted biannually providing an opportunity to learn and improve their basic surgical skills. These skills taught during workshops were basic instrument handling skills, suturing techniques, hand tying, urethral catheterization, back slab application, chest tube insertion, nasogastric tube insertion, gowning and gloving, the method of using a hand-held doppler, wound care and the method of performing a sterile dressing in the simulation lab. 

In the end, the overall satisfaction level for the internship program was assessed. The proportion of interns satisfied with the program was significantly higher in phase 2 (75.4% vs 50%, p=0.02) and the mean rank for overall satisfaction was also higher in phase 2 (74.9 vs 60.3, p=0.02).

## Discussion

Maintaining high job satisfaction while facilitating work-based training for a diverse group of young medical graduates is a huge challenge [3,14]. Like a few other countries, in Pakistan an internship, also known as a house job, is a mandatory one-year training period, which commences immediately after graduation from medical school. It is considered as a prerequisite for awarding an MBBS degree, allowing a medical graduate to practice or join a formal residency program [12]. The switch from classrooms to a work-based environment with responsibilities is a challenging task for young graduates which if not facilitated efficiently may lead to burnout and producing doctors with compromised skills and compassion. The current study is the first from Pakistan reporting our experience of the restructuring internship program aiming to improve intern’s on-the-job learning and satisfaction in a university hospital setting.

Annually 76 interns are enrolled after a merit-based, competitive evaluation process of written exam and OSCE with approximately more than 500 applicants from all over the country having diverse academic backgrounds. Based on an elaborate external review conducted in 2012, the Internship Committee at AKUH revamped its program, adopting the six-step approach described by Kern as an instructional design process [12]. Like other studies, a mixed model approach was adopted to evaluate satisfaction levels of stakeholders as well as an improvement in the intern's satisfaction with work-based training while allowing participants for their anonymous, spontaneous feedback without any restriction [3,15]. Rated as a program worth joining due to the high impact of the institution with training facilities matching international standards, structured programs with a secure workplace and good salary structure were reassuring.

Our results supported the fact that introducing fundamental changes to an existing program can improve overall satisfaction for a work-based training program. The role of regulating working hours and introducing mentoring opportunities has been shown to improve job satisfaction, and our results further endorse this notion [9,10]. The mentorship opportunities introduced consisted of regular career counseling sessions (individuals and groups), quarterly Intern’s forum to discuss their issues in an open, confidential, and friendly atmosphere. The interns were further allowed easy access to individual faculty members including Program Director, Coordinators, and Associate Dean to discuss their issues.

Restructuring of orientation program with exposure in clinical areas under supervision provided an opportunity to familiarize interns with the existing systems, policies operating mechanisms, safe handovers, and daily working mechanisms. With the implementation of the 12-month rotational plan, interns were able to select rotations of their choice. Sharing of learning objectives of all rotations beforehand helped the interns to focus on what they can learn during a specific rotation. The restructuring of mandatory teaching sessions and topics covered found useful. These results were consistent with existing evidence. For instance, sharing a checklist of expected competencies and desired objectives at the start of surgical rotation was found to be associated with better performance and satisfaction levels [16]. However, we feel there is still more room to improve the orientation program and the diversification of rotations. Compliance with achieving objectives of rotations in a challenging working environment is still an area to work on. The content-based knowledge also needs revision to improve the utility.

In a busy tertiary care hospital with multiple learners including fellows, residents, interns, students creating equal opportunities for all and supervision by faculties at the workplace is difficult and this is what interns have reported in our study as well [14]. However, after restructuring, significant improvement in satisfaction level with hands-on experience and supervision was observed in inpatient areas and operating rooms but not in clinics. Peer learning and supervision were greater with fellows as compared to faculties reflecting the need for further measures to improve. These results are consistent with other studies and the most probable reason is the parallel provision of services and teaching at the workplace [14]. 

Surgical skills workshop, in particular, not only allowed an opportunity for interns to learn the basics of surgical procedures but also a close interaction with surgical faculty, which helped to improve their work environments. Following the same strategy, periodic workshops to enhance other competencies including communication skills are expected to improve the desired outcome.

The internship year is unique, bridging between two immensely established, structured, curriculum-based programs i.e. undergraduate and postgraduate medical education. While providing opportunities to groom in challenging patient-centered workplace, rotations in various subspecialties help them to choose a further course of their careers [17,18]. Most of the interns also prepare for the internship exit exam as well as an entry exam for residency during the internship adding further stress. Often the busy patient-centered systems, presence of multiple learners allow little time for faculty interaction with interns leading to lack of a sense of ownership and dissatisfaction for interns. Developing mechanisms to improve compliance with implemented strategies, working hours and mentorship, additional measures including protected time for study, supervision with immediate seniors like residents as well as faculties are expected to achieve the desired outcome. Additionally, timely feedbacks from all stakeholders would help to streamline the way forward. Although no change has been observed in satisfaction level for many aspects, our study reported substantial improvement in major areas and overall satisfaction even with minor evidence-based adjustments to the internship program. Being single centered and smaller sample, our study does carry limitations. In the future planning audits with a larger sample and objective parameters to measure outcome will provide a roadmap.

## Conclusions

Our study was thus able to conclude that while the internship year is extremely stressful and demanding, several interventions can be put into place to improve work-based learning and satisfaction among interns thus improving their experience and performance. In this two-phase study, in light of the responses received in the initial round, we were able to effectively restructure different aspects of the internship for the second phase, thereby significantly increasing intern satisfaction and learning. Thus the regular audits, feedback from all stakeholders, and adopting strategies to improve an academic program will help to achieve the desired outcomes. These attempts subsequently enable the interns to become more competent and well-rounded health practitioners.
